# Prediction of all-cause and cardiovascular mortality using central hemodynamic indices among elderly people: systematic review and meta-analysis

**DOI:** 10.1590/1516-3180.2020.0364.r1.0412020

**Published:** 2021-03-12

**Authors:** Tarsila Vieceli, Bárbara Brambilla, Raphael Quintana Pereira, Bruno Schmidt Dellamea, Airton Tetelbom Stein, Guilherme Brasil Grezzana

**Affiliations:** I MD, MSc. Internal Medicine Resident, Hospital de Clínicas de Porto Alegre, Porto Alegre (RS), Brazil.; II Medical Student, Universidade de Caxias do Sul (UCS), Caxias do Sul (RS), Brazil.; III MD. Cardiovascular Surgeon, Hospital Infantil Dr. Jeser Amarante Faria, Joinville (SC), Brazil.; IV MD, PhD. Professor, Universidade de Caxias do Sul (UCS), Caxias do Sul (RS), Brazil.; V MD, PhD. Professor, Universidade Federal de Ciências da Saúde de Porto Alegre (UFCSPA), Porto Alegre (RS), Brazil.; VI MD, PhD. Cardiologist, Clínica Del Cuore, Antônio Prado (RS), Brazil.

**Keywords:** Cardiovascular diseases, Aged, Preventative health services, Cardiovascular, Geriatric cardiology, Preventive health care

## Abstract

**BACKGROUND:**

Despite widespread usage of central blood pressure assessment its predictive value among elderly people remains unclear.

**OBJECTIVE:**

To ascertain the capacity of central hemodynamic indices for predicting future all-cause and cardiovascular hard outcomes among elderly people.

**DESIGN AND SETTING:**

Systematic review and meta-analysis developed at the Del Cuore cardiology clinic, in Antonio Prado, Rio Grande do Sul, Brazil.

**METHODS:**

312 full-text articles were analyzed, from which 35 studies were included for systematic review. The studies included needed to report at least one central hemodynamic index among patients aged 60 years or over.

**RESULTS:**

For all-cause mortality, aortic pulse wave velocity (aPWV) and central systolic blood pressure (SBP) were significant, respectively with standardized mean difference (SMD) 0.85 (95% confidence interval, CI 0.69-1.01; I^2^ 96%; P < 0.001); and SMD 0.27 (95% CI 0.15-0.39; I^2^ 77%; P 0.012). For cardiovascular mortality brachial-ankle PWV (baPWV), central SBP and carotid-femoral PWV (cfPWV) were significant, respectively SMD 0.67 (95% CI 0.40-0.93; I^2^ 0%; P 0.610); SMD 0.65 (95% CI 0.48- 0.82; I^2^ 80%; P 0.023); and SMD 0.51 (95% CI 0.32-0.69; I^2^ 85%; P 0.010).

**CONCLUSIONS:**

The meta-analysis results showed that aPWV was promising for predicting all-cause mortality, while baPWV and central SBP demonstrated consistent results in evaluating cardiovascular mortality outcomes. Thus, the findings support usage of central blood pressure as a risk predictor for hard outcomes among elderly people.

**REGISTRATION NUMBER IN PROSPERO:**

RD42018085264

## INTRODUCTION

Brachial arterial blood pressure is still widely used as a predictive parameter for cardiovascular damage, morbidity and mortality, in assessing cardiovascular risk within clinical practice. However, this does not correspond to central blood pressure measured through the carotids and ascending aorta.^1^^,^^2^ Previous studies have demonstrated that central blood pressure measurements are better predictors of vascular disease and cardiovascular events than brachial pressure.^3^^–^^5^

Central blood pressure relates to arterial stiffening and the aging process, and it is an independent predictor for cardiovascular clinical events.^6^ Furthermore, the pharmacological superiority of vasodilating drugs with regard to cardiovascular outcomes may be due to their different effects on central blood pressure, rather than similar effects on brachial blood pressure.^7^ Thus, peripheral blood pressure measurements may not be a proper substitute for assessing the antihypertensive effects of arterial hemodynamics.^8^

Despite the relevance of central hemodynamic measurement in making diagnoses, determining therapies and making prognoses, many aspects of these measurements remain unclear. This lack of clarity is reflected in low usage of this method in clinical practice. The definition of cutoff values for central blood pressure varies between different ages and populations,^9^ especially in older populations, whose distinct aging and pathological stiffening of arteries may constitute confounding factors with regard to central arterial hypertension. One confounding factor is that either indications for central blood pressure assessment are absent from guidelines^10^ or, when present, their use has been shown to only have questionable incremental value for diagnosing hypertension, compared with standard arterial pressure, except in assessing systolic arterial pressure in young adults.^2^

It has been shown that central blood pressure assessment is widely used as a substitute marker for predicting future cardiovascular events.^7^ Nonetheless, the predictive value of this marker in populations that are known to be susceptible, like the elderly, remains unclear in the data in the literature.

## OBJECTIVE

Thus, the present systematic review and meta-analysis was conducted with the aim of providing a quantitative estimate of the capacity of central blood pressure for predicting future cardiovascular events in older populations. In addition, the aim was to assess the current pending issues regarding the applicability of this method. Through this, it was sought to glean evidence to support usage of indirect central blood pressure assessment within daily clinical practice.

## METHODS

### Protocol and registration

This systematic review was reported in accordance with the MOOSE guidelines (Meta-analysis Of Observational Studies in Epidemiology).^11^ Additionally, we took into account the guidelines of PRISMA (Preferred Reporting Items for Systematic Reviews and Meta-Analysis)^12^ and AMSTAR 2 (A MeaSurement Tool to Assess Reviews).^13^ The protocol for this study was registered in the Prospero database (International Prospective Register of Systematic Reviews),^14^ under the code CRD42018085264, and this protocol was previously published in a scientific journal.^15^

### Eligibility criteria

We included full peer-reviewed articles that reported on longitudinal studies that had included samples of patients with a mean age of 60 years or over. The studies included needed to have reported at least one of the following central hemodynamic indexes: central systolic blood pressure (SBP), central pulse pressure (PP), central augmentation index (AIX), aortic pressure, wave reflection (WR) and pulse wave velocity (PWV). Additionally, the studies included needed to have reported all-cause mortality and/or cardiovascular mortality as the outcome. We excluded studies if they had reported results from duplicate populations.

### Information sources

We searched the following electronic databases: PubMed, EMBASE and Virtual Health Library (VHL), which contained citations from LILACS, IBECS, MEDLINE, Cochrane Library and SciELO. In addition, we manually searched the reference lists of the articles included and performed citation analysis on the studies included, using Google Scholar. We also sought experts’ suggestions through e-mail communications.

### Search

The initial search comprised the MeSH terms “Aged”, “Aged, 60 and over”, “Pulse wave analysis” and related entry terms, along with other terms relating to central hemodynamics such as “Central systolic blood pressure”, “Central pulse pressure”, “Central augmentation index”, “Central pressures”, “Aortic pressure”, “Wave reflections”. A sensitive search strategy for observational studies was also used. The complete search strategy used for the PubMed database is shown in **Appendix 1.** We did not impose any limits for language.

### Study selection

The titles and abstracts of the articles retrieved were independently evaluated by two reviewers (GC, TV). Abstracts that did not provide enough information regarding the eligibility criteria were kept for full-text evaluation. The reviewers independently evaluated the full-text articles and determined study eligibility. Any disagreements were resolved through reaching a consensus among three other researchers (GBG, ATS, CR).

### Risk of bias

Risk of bias was evaluated by ranking each study in accordance with the ROBINS-I tool (Risk Of Bias in Non-randomized Studies - of Intervention).^16^ The following types of bias were considered: bias due to confounding, bias in selection of participants into the study, bias in classification of interventions, bias due to deviations from intended interventions, bias due to missing data, bias in measuring of outcomes, bias in selection of the reported result and overall bias. Each item was classified as presenting low, moderate, serious or critical risk of bias, or as “no information” when the article did not provide any information on which to base a judgement about risk of bias for this domain. This evaluation was performed independently by two reviewers (GC, TV).

Any disagreements were resolved through reaching a consensus with two other researchers (GBG, ATS).

### Data extraction

Four reviewers independently conducted data extraction, and any disagreements were resolved through reaching a consensus among three other researchers. Data on the general characteristics of the studies were collected, such as: study title, author, journal and year of publication, study design, inclusion and exclusion criteria, outcomes definitions, outcome measurements and follow-up. In addition, we extracted specific information about central hemodynamic indexes and their predictive values (when available).

### Data analysis

The data collected were extracted to the Microsoft Excel software v16.42 (Microsoft, Redmond, United States) for tabulation. The meta-analyses were performed using the STATA software v11.0 (STATA Corp., College Station, United States).

The meta-analyses was made using a fixed model. To analyze the methods used for measuring central pressure hemodynamics, we used the standardized mean difference (SMD) for quantitative variables. We used P (Peeta) and I² to assess heterogeneity, Egger's test and Begg's test for small study biases and funnel plot graphs for publication biases. Trim-and fill analyses were performed to validate the data. The final results were presented using a forest plot graph. Meta-regression and sensitive analyses were evaluated for confounding biases.

## RESULTS

**Figure 1:**
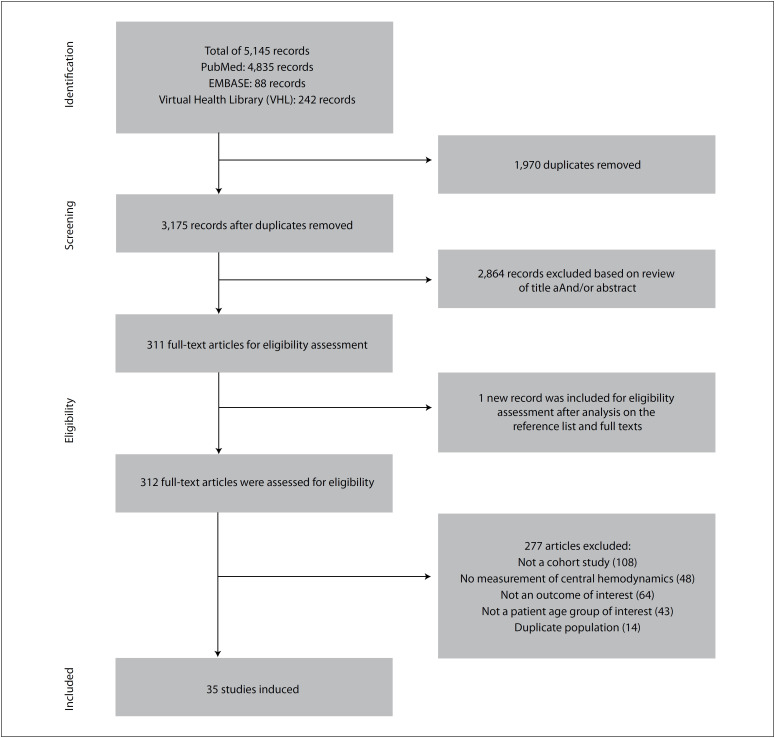
Study selection flow diagram.

Our search strategy yielded a total of 5,145 citations from electronic databases. After the keyword and medical term search, we included 5,145 abstracts for review. After removing duplicates and excluding records based on analysis of their titles, 312 full-text articles were analyzed, from which 35 studies were included for systematic review. **Figure 1** presents the study selection flow diagram.

**Figure 2:**
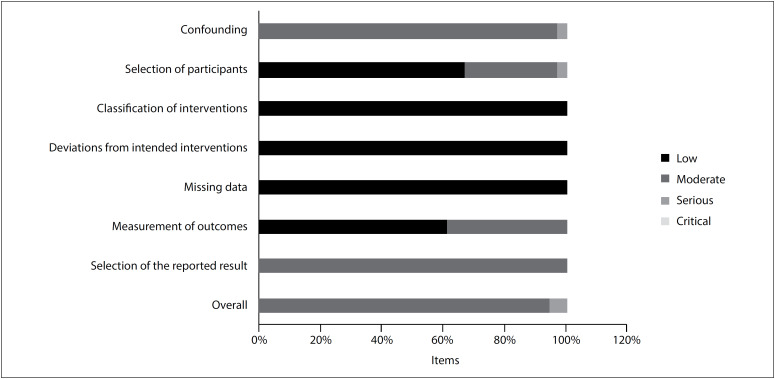
Risk-of-bias evaluation of methodological quality, in accordance with the ROBINS-I tool (Risk Of Bias In Non-randomized Studies - of Interventions).

The studies included were published between 2001 and 2016. Out of these 35 studies, 32 (91%) were prospective cohorts and three (9%) were retrospective cohorts. The total participants comprised patients with diabetes (3.9%), hemodialysis patients (10.29%), and patients receiving coronary interventions (2.6%). The participants’ mean ages ranged from 60 ± 11 years to 86.8 ± 6.9 years. The prevalences of hypertension and diabetes ranged from 19% to 100% and from 7.1% to 100%, respectively. The prevalence of histories of cardiovascular disease ranged from 3.1% to 60.2%; the most prevalent events were stroke and myocardial infarction. A more detailed overview of the study characteristics is available in **Table 1**. All the studies were assessed for methodological quality in accordance with the ROBINS-I tool (Risk Of Bias in Non-randomized Studies - of Interventions), and these assessments are presented in **Figure 2**. The risk-of-bias evaluation on each study included is shown in further detail in **Table 2** and is summarized in **Figure 2**.

**Table 1:** Studies included in the meta-analysis

Study, year	Methodevaluated	Outcome	Population	Mean age(mean ± SD)	Malegender(%)	Hypertension(%)	Diabetes(%)	BMI(mean ± SD)	History of CVD(%)
Anderson, 2009^34^	aPWV	All-cause	Nondiabetic participants aged 45 to 74 years	Arm 1: 58.7 (57-59) Arm 2: 63.6 (62.1-65.1)*	Arm 1: 49.1 Arm 2: 55.1	Arm 1: 19 Arm 2: 16^&^	NA	Arm 1: 26.6 (25.9-27.4) Arm 2: 25.7 (24.7-26.7)*	NA
Cruickshank, 20 02^32^	aPWV	All-cause	Patients with DM2	60 (59-71)*	57.6	NA	100	NA	NA
Huang, 2011^6^	CSBP	All-cause, CV	Patients receiving PCI	70 ± 12	88	66	34	NA	MI: 17 CABG/PCI: 30
Kato, 20 1 0^43^	baPWV	CV	Patients who had been undergoing regular HD	64 ± 12	65.4	NA	20.1	NA	20.1
Kato, 2012^36^	baPWV	CV	Patients undertaking regular HD	60 ± 11	67		36.4		12.5
Meaume, 2001^48^	cfPWV	CV	Patients hospitalized for rehabilitation after infectious disease, CHF, recent surgery, recent stroke, or end-stage Parkinson's disease.	87.1 ± 6.6	27	NA	NA	22.03 ± 3.97	Atherosclerosis of the lower limbs: 16 Previous stroke: 21 Previous MI: 12
Onuigbo, 2013^33^	aPWV	All-cause	Patients undergoing regular HD	NA	Arm 1: 45.8 Arm 2: 57.8	NA	NA	NA	NA
Pini, 2008^5^	CSBP	CV	Community-dwelling individuals ≥ 65 years of age	73 ± 6	45		9	26.7 ± 4.3	Stroke, TIA: 5 PVD: 10 CAD: 9
Sung, 2011^21^	AIX	All-cause	Patients with acute heart failure syndrome	Arm 1: 72.2 ± 14.9 Arm 2: 75.0 ± 12.5	Arm 1: 82.4 Arm 2: 82.8	Arm 1: 74.5 Arm 2: 82.8	Arm 1: 39.2 Arm 2: 58.6	Arm 1: 25.6 ± 5.1 Arm 2: 24.1 ± 4.2	Arm 1: 51 Arm 2: 62.1
Tziomalos, 2014^20^	aPWV, CSBP, AIX, cPP	All-cause	Patients who were admitted with acute ischemic stroke	Arm 1: 81.9 ± 7.6 Arm 2: 78.5 ± 6.5	Arm 1: 47.2 Arm 2: 38.5	Arm 1: 72.2 Arm 2: 83.9	Arm 1: 30.5 Arm 2: 32.7	Arm 1: 27.1 ± 6.4 Arm 2: 27.3 ± 4.9	CAD: Arm 1 27.7; Arm 2 27.9. Previous stroke: Arm 1 44.4; Arm 2 41.2
Van Sloten, 2014^19^	AIX, cfPWV	All-cause, CV	Population- based cohort in the Netherlands	Arm 1: 69.0 ± 6.4 Arm 2: 71.9 ± 6.2	50 overall Arm 1: 45.2 Arm 2: 64.6	Arm 1: 62.9 Arm 2: 80.6	23 overall Arm 1: 22.1 Arm 2: 25.8	Arm 1: 27.0 ± 3.6 Arm 2: 27.0 ± 3.4	Arm 1: 47 Arm 2: 63.8
Zhang, 2013^22^	CSBP, cPP	All-cause	Hospitalized elderly patients	86.8 ± 6.9	25.98	75.2	20.9	27.2 ± 5.7	CHD: 33 HF: 22.2 AF: 17.2

* Data are presented as mean (range); % - data only available for men and women separately; ^&^Only patients receiving anti-hypertensive drug therapy were considered hypertensive.

NA = data not available; MI = myocardial infarction; PCI = percutaneous coronary intervention; CABG = coronary artery bypass grafting; SBP = systolic blood pressure; DBP = diastolic blood pressure; aPWV = aortic pulse wave velocity; CSBP = central systolic blood pressure; AIX = augmentation index; cPP = central pulse pressure; baPWV = brachial-ankle pulse wave velocity; cfPWV = carotid-femoral pulse wave velocity; PWV = pulse wave velocity; CV = cardiovascular; DM2 = type 2 diabetes mellitus; CHF = chronic heart failure; HF = heart failure; HD = hemodialysis; CAD = coronary artery disease; AF = atrial fibrillation;

TIA = transient ischemic attack; PVD = peripheral vascular disease.

**Table 2:** Meta-analysis results

All-cause mortality						
Method	Studies	Year	Cases	Controls	SMD (±95% CI)	Weight (%)
aPWV	Anderson et al.^34^	2009	60	114	2.34 (1.94 to 2.74)	16.25
	Cruickshank et al.^32^	2002	22	97	1.83 (1.32 to 2,35)	9.58
	Onuigbo et al.^33^	2013	106	308	0.40 (0.17 to 0.62)	52.22
	Tziomalos et al.^20^	2014	36	379	0.41 (0.07 to 0.76)	21.95
	Overall (I-squared = 96.7%; P = 0.000)				0.85 (0.69 to 1.01)	100
CSBP	Huang et al.^6^	2010	201	813	0.42 (0.27 to 0.58)	59.92
	Tziomalos et al.^20^	2014	36	379	0.09 (-0.25 to 0.43)	12.39
	Zhang et al.^22^	2013	110	221	0.03 (-0.19 to 0.26)	27.69
	Overall (I-squared = 77.4%; P = 0.012)				0.27 (0.15 to 0.39)	100
AIX	Sung et al.^21^	2011	29	51	0.26 (-0.19 to 0.72)	14.08
	Tziomalos et al.^20^	2014	36	379	-0.90 (-1.25 to −0.55)	24.46
	Van Sloten et al.^19^	2014	96	483	0.11 (-0.11 to 0.33)	61.46
	Overall (I-squared = 92.4%; P = 0.000)				-0.11 (-0.29 to 0.06)	100
cPP	Tziomalos et al.^20^	2014	36	379	-0.57 (-0.91 to −0.22)	30.66
	Zhang et al.^22^	2013	110	221	0.06 (-0.17 to 0.29)	69.34
	Overall (I-squared = 88.6%; P = 0.003)				-0.13 (-0.33 to 0.06)	100

Cardiovascular mortality						
Method	Studies	Year	Cases	Controls	SMD (±95% CI)	Weight (%)
baPWV	Kato et al.^43^	2010	39	156	0.61 (0.25 to 0.96)	56.11
	Kato et al.^36^	2012	33	102	0.75 (0.34 to 1.15)	43.89
	Overall (I-squared = 0.0%; P = 0.611)				0.67 (0.40 to −0.93)	100
CSBP	Pini et al.^5^	2008	122	276	0.50 (0.28 to 0.72)	62.10
	Huang et al.^6^	2011	55	813	0.91 (0.63 to 1.18)	37.90
	Overall (I-squared = 80.6%; P = 0.023)				0.65 (0.48 to 0.82)	100
cfPWV	Meaume et al.^48^	2001	73	68	0.15 (-0.18 to 0.48)	31.14
	Van Sloten et al.^19^	2014	96	483	0.67 (0.45 to 0.89)	68.86
	Overall (I-squared = 85%; P = 0.010)				0.51 (0.32 to 0,69)	100

aPWV = aortic pulse wave velocity; CSBP = central systolic blood pressure; AIX = augmentation index; cPP = central pulse pressure; baPWV = brachial-ankle pulse wave velocity; cfPWV = carotid-femoral pulse wave velocity; SMD = standardized mean difference; CI = interval confidence.

### All-cause mortality

#### Augmentation index

Five studies used the augmentation index (AIX). In two of these studies, the population consisted of patients who had undergone coronary angiography^17^^,^^18^ (the increase in AIX@75 was correlated with the mortality events). In one study,^17^ the population was formed only by male patients. The populations of the remaining studies comprised one population-based cohort^19^ (AIX@75 was not correlated with the mortality events), one group of patients experiencing acute ischemic stroke^20^ (which was associated with intra-hospital mortality) and one group of patients with heart failure^21^ (which was shown to have significant predictive value regarding mortality post-hospital discharge).

#### Augmented pressure

One study^21^ evaluated carotid augmented pressure (cAP) in patients hospitalized due to heart failure and found that cAP showed a significant association with all-cause mortality. Additionally, a higher risk of all-cause mortality was observed in another study, among patients who underwent coronary angiography.^17^

#### Central systolic blood pressure

Central systolic blood pressure assessment was used in three studies.^6^^,^^20^^,^^22^ In a cohort studied by Tziomalos et al.,^20^ central SBP did not present any association with the outcome, compared with patients who received hospital discharge. A study on a population with previous histories of cardiovascular disease^22^ did not demonstrate any association with the outcome. However, in a study on decompensated heart failure patients who had been admitted to the emergency ward,^21^ the findings were significant as a predictive value regarding post-hospital discharge.

#### Central pulse pressure

Central pulse pressure (cPP) was evaluated as a marker of arterial stiffness in three studies.^20^^–^^22^ In a study on patients with acute ischemic stroke (AIS), the lowest cPP values were associated with intra-hospital mortality.^20^ In patients with decompensated heart failure,^21^ cPP presented significant predictive values for all-cause mortality post-hospital discharge. In a study on hospitalized elderly patients with histories of cardiovascular disease,^22^ increased cPP was not associated with mortality.

#### Carotid-femoral pulse wave velocity (cfPWV) and aortic pulse wave velocity (aPWV)

Carotid-femoral PWV (cfPWV) was used to measure central arterial stiffness in 12 studies. In one study, higher baseline cfPWV levels were associated with all-cause mortality for patients with estimated glomerular filtration rate (eGRF) < 90 ml/min/1.73 cm^2^.^23^ In a study on subjects undergoing hemodialysis,^24^ cfPWV was a significant predictor of events within six months after discharge. However, in a study by Hoom et al., higher cfPWV in patients with higher risk of cardiovascular events was not predictive for all-cause mortality.^19^ In a cohort of inpatients, there was no association with mortality.^22^ In the Rio de Janeiro Type 2 Diabetes Cohort Study, separate analyses for cardiovascular and non-cardiovascular mortality did not show any association.^25^

In a cohort of very old frail subjects with histories of cardiovascular diseases,^26^ a positive nonsignificant trend was observed between PWV and mortality risk. In a study that also evaluated hemodialysis patients,^27^ the odds ratios were 1.3 for PWV-high/low and 3.2 for PWV high/high, compared with the PWV-low/low reference group.

In a population with decompensated heart failure, cfPWV had significant predictive values for adverse post-discharge outcomes.^21^ In a cohort of Japanese patients, the Kaplan-Meier time-to-event curves for death from all causes differed significantly among the four groups over the entire follow-up period (P < 0.0001).^28^

In the Rotterdam Study, a trend in relation to all-cause mortality was observed after data adjustment.^29^ In well-functioning community-dwelling subjects, the association between all-cause mortality and PWV was not independent of heart rate.^30^ Lastly, in a prospective cohort of Japanese-Americans, higher PWV values were significantly associated with all-cause mortality. However, multivariate analysis revealed that there was only a higher tendency towards all-cause mortality, which was not statistically significant.^31^

The parameter of aortic PWV measurement was used in four cohort studies to estimate central arterial stiffness.^20^^,^^32^^–^^34^ In patients with type 2 diabetes (DM2), PWV was an independent predictor of later mortality across the entire spectrum of glucose tolerance.^32^ In a population undertaking regular hemodialysis, the baseline PWV was lower for survivors than for dead patients.^33^ In a study on a nondiabetic population, PWV had a significant independent impact on all-cause mortality.^34^ In a cohort of AIS patients, on the other hand, aortic PWV did not show any predictive value for the all-cause mortality outcome. **Table 1** summarizes all the studies reporting all-cause and cardiovascular mortality.^20^

#### Brachial-ankle pulse wave velocity (baPWV)

Ten studies used baPWV to evaluate arterial stiffness in patients older than 60 years.^35^^–^^44^ In a cohort of diabetic patients, baPWV values were a significant predictor of the mortality endpoint.^39^ In a study on patients with lacunar stroke syndrome, those with high baPWV values were at higher risk of all-cause mortality.^40^ In an Asian study on patients in the acute phase of stroke, patients with higher baPWV were at higher risk of all-cause mortality.^41^ In a review study that included patients with DM2, the combination of ankle brachial index (ABI) and baPWV showed significantly higher all-cause mortality rates.^42^ In another cohort, after multivariate hazard ratio (HR) analysis, the results showed a significant difference between the top decile of baPWV and the whole study population for all-cause mortality outcomes.^35^

In four studies, patients were undergoing hemodialysis were evaluated.^36^^,^^38^^,^^43^^,^^44^ In a study by Kato et al.,^43^ patients with baPWV values in the highest tercile had a significantly lower survival rate than those in the middle and lowest terciles.^43^ In a later study, the total survival rate was significantly lower among patients with higher baPWV.^36^ In a retrospective cohort study, baPWV was a significant predictor of the all-cause mortality outcome.^44^ Additionally, in the Kahoku longitudinal study, higher baPWV levels were significantly associated with all-cause mortality.^38^

### Cardiovascular mortality

#### Brachial-ankle pulse wave velocity (baPWV)

In six studies, baPWV was used to evaluate arterial stiffness and the corresponding predictive value for cardiovascular mortality. In a Chinese population, baPWV was significantly associated with cardiovascular mortality.^35^ In a cohort of hemodialysis patients, no increase in baPWV was observed.^45^ In another study on a population undergoing hemodialysis, patients with higher baPWV presented higher cardiovascular mortality risk than those in the lower terciles.^36^ In a longitudinal study, high baPWV levels were significantly associated with higher risk of three-year cardiovascular mortality.^37^ In a Japanese cohort that observed 85 endpoints, cardiovascular mortality was progressively and significantly greater from the second quartile of baPWV onwards.^38^ Lastly, in the LILAC study, the increase in baPWV was associated with an increased risk of cardiovascular mortality.^46^

#### Carotid-femoral pulse wave velocity (cfPWV)

Four studies used cfPWV.^30^^,^^31^^,^^47^^,^^48^ In a hemodialysis patient cohort,^47^ increasing terciles of PWV1 but not those of PWV2 or PWV3 were significantly correlated with cardiovascular mortality. A prospective study on Japanese-Americans showed that higher PWV values correlated with higher risk of cardiovascular mortality.^31^ Another study showed significant associations with cardiovascular mortality.^30^ A further study was conducted on subjects over 70 years of age and it was found that increased PWV was associated with cardiovascular mortality.^48^

#### Diverse parameters for pulse wave velocity evaluation

In a cohort study on patients undergoing hemodialysis, the prognostic value of cfPWV, carotid AI, CPP and carotid-brachial pulse pressure amplification (AMP) were measured. The AI, CPP and AMP parameters after dialysis did not show any association with cardiovascular mortality.^49^ In another cohort, the carotid SBP was an independent predictor of cardiovascular mortality after eight years of follow-up.^5^ In a study on patients who underwent percutaneous coronary intervention, CPP itself was independently associated with the risk of cardiovascular events after this procedure.^50^

### Meta-analysis

**Table 1** provides a description of all the studies used in the meta-analysis, grouped according to the methods used: PWV, central SBP, AIX and cPP. The results regarding all-cause mortality and cardiovascular mortality are presented in **Table 2**.

For all-cause mortality, we found 28 studies, among which eight were included in the meta-analysis. Three of these evaluated AIX, three evaluated central SBP, two evaluated cPP and four evaluated aPWV. There were 878 cases in total, and 3,824 controls. Among the remaining studies, either the data were not shown or it was not feasible to extract the data.

For cardiovascular mortality we found 20 studies, among which six were included in the meta-analysis. These comprised two evaluating central SBP, two evaluating baPWV and two evaluating cfPWV, with a total of 418 cases and 1898 controls. Among the remaining studies, either the data were not shown or it was not feasible to extract the data.

**Figure 3:**
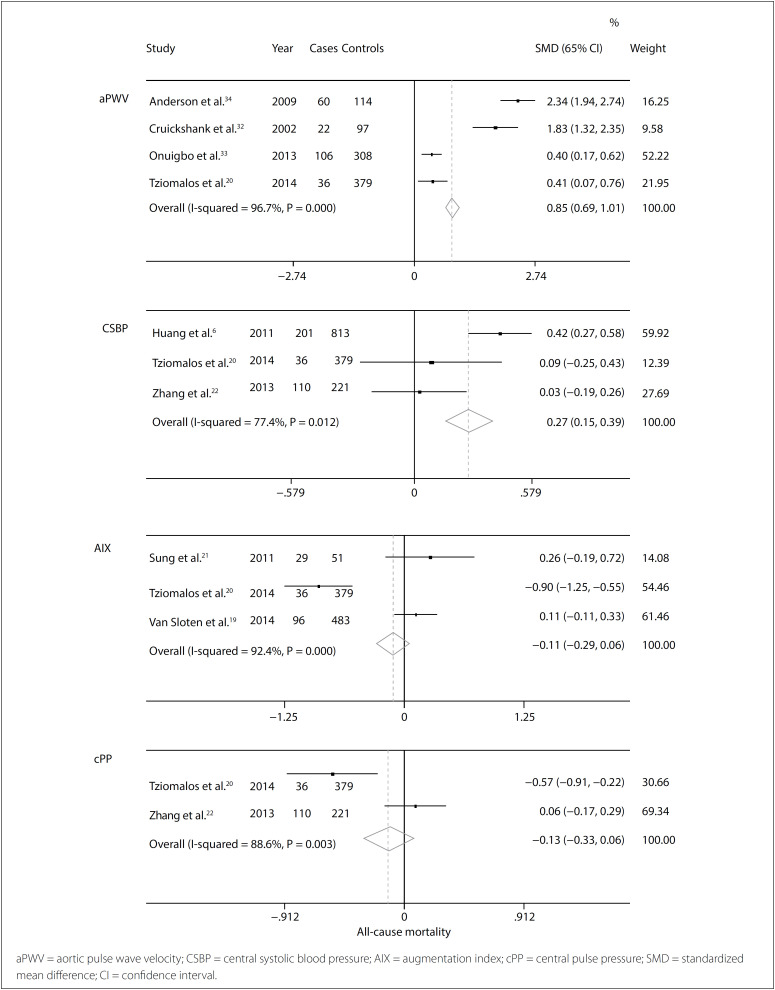
Forest plot for all-cause mortality and indirect central blood pressure assessment method.

For all-cause mortality, aPWV and central SBP were significant, respectively with SMD 0.85 (95% CI 0.69-1.01; I^2^ 96%; P < 0.001) and SMD 0.27 (95% CI 0.15-0.39; I^2^ 77%; P 0.012). Also for all-cause mortality, AIX and cPP were not significant, respectively with SMD −0.11 (95% CI −0.29-0.06; I^2^ 92%; P < 0.001) and SMD −0.13 (95% CI −0.33-0.06; I^2^ 88%; P 0.003) (**Figure 3**).^6^^,^^19^^–^^22^^,^^32^^–^^34^

**Figure 4:**
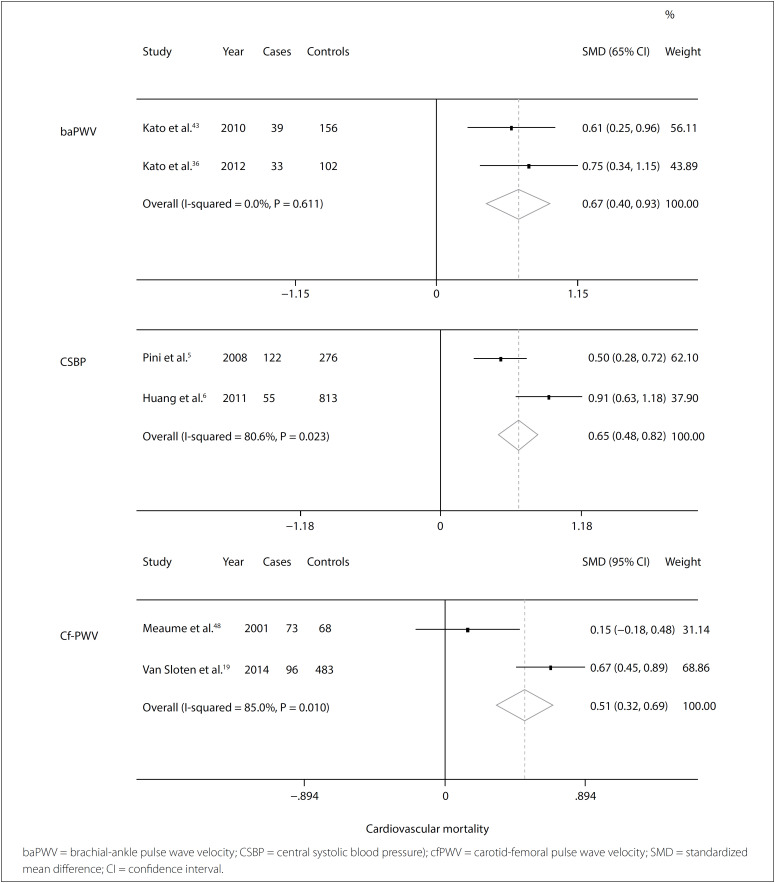
Forest plot for cardiovascular mortality and indirect central blood pressure assessment method.

For cardiovascular mortality, baPWV, central SBP and cfPWV were significant, respectively with SMD 0.67 (95% CI 0.40-0.93; I^2^ 0%; P 0.610), SMD 0.65 (95% CI 0.48-0.82; I^2^ 80%; P 0.023) and SMD 0.51 (95% CI 0.32-0.69; I^2^ 85%; P 0.010) (**Figure 4**).

**Figure 5:**
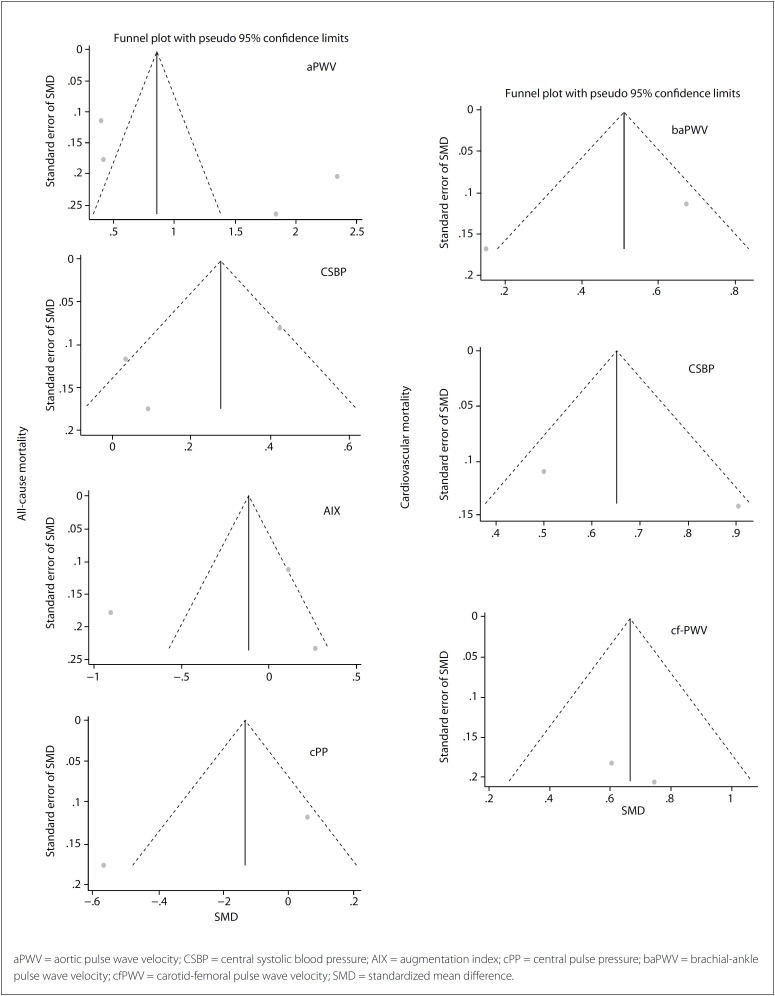
Funnel plot for all-cause mortality and cardiovascular mortality for each indirect central blood pressure assessment method.

The evaluation on biases is shown in **Figure 5**, as funnel plots for all-cause mortality and cardiovascular mortality. Begg's test and Egger's teste were performed. Fill-and-trim analyses were performed for aPWV in relation to all-cause mortality, and for central SBP and baPWV in relation to cardiovascular mortality, and these showed that there was no modification of the results, with P < 0.001 for all of these analyses. In relation to age and sex prevalence, we performed a meta-regression that showed that the variable aPWV did not make any contribution to all-cause mortality. **Table 2** summarizes the bias evaluations.

## DISCUSSION

In our study, increased aortic PWV and central SBP were associated with all-cause mortality. Higher baPWV, central SBP and cfPWV were associated with cardiovascular mortality, with higher accuracy for prediction, compared with the parameters for peripheral arterial pressure evaluation. Central blood pressure was found to reflect arterial stiffness and hemodynamic pressure in the heart and great vessels more accurately for predicting intermediate and surrogate endpoints, compared with brachial arterial pressure.^25^

Arterial stiffness increases with aging, and it has been suggested that this is a modulator of atherosclerosis progression and hypertension.^41^ Although central blood pressure is used as a marker for clinical outcomes, few studies have evaluated the possibility of validating this prognostic tool for older populations.^26^ Therefore, it was sought through the present systematic review and meta-analysis to evaluate the evidence regarding usage of central pressure data, in various assessment techniques, as a predictor of hard clinical outcomes in older populations. The main conclusion reached was that in all the studies eligible for the meta-analysis, central pressure data presented predictive value for mortality and cardiovascular outcomes. Moreover, among these studies, two studies showed that central blood pressure was predictive of all-cause mortality, regardless of the non-invasive technique used for its measurement. These results give rise to the possibility of differentiating physiological vascular aging in older patients according to their biological alterations and the chronic structures that unleash the pathological process of arterial stiffness and its consequent clinical implications.

This strong relationship between central blood pressure findings and cardiovascular mortality suggest that there is a close relationship between arterial stiffness and traditional cardiovascular risk factors. Alterations to homeostasis, with reduction of coronary diastolic filling, plus elastic abnormalities of the aorta due to arterial stiffness, as observed through raised PWV,^26^ is a plausible mechanism that would contribute to the observed outcomes. The studies reviewed here show that it is feasible to make fast non-invasive PWV measurements with reliable results and that these could become part of routine outpatient clinical care.

The two techniques for estimating central arterial stiffness that demonstrated predictive value both for all-cause mortality and for cardiovascular mortality were aortic PWV (aPWV) and central SBP. aPWV was an independent predictor of subsequent all-cause and cardiovascular mortality among patients with or without DM2 and among patients with chronic kidney failure. However, in a cohort of patients with acute ischemic stroke,^20^ aPWV did not present any predictive value for all-cause mortality. Central SBP was also a predictor of all-cause mortality among patients with heart failure after hospital discharge.^21^ This observation raises the possibility of using central blood pressure measurement as a therapeutic orientation within this clinical context, considering that a larger clinical benefit seems to be achieved through a large decline in central blood pressure.^20^ Studies that used baPWV were also selected in this review. baPWV is an indicator of the combination of central and peripheral arterial stiffness, and previous longitudinal studies and a meta-analysis have demonstrated that the prognostic value of baPWV is as significant as the value of cfPWV.^51^

The population in the studies selected here (i.e. older patients) was theoretically less susceptible to the effects of arterial stiffness with later clinical manifestation. However, one of the factors that may have contributed to the findings in the present study was the presence of high numbers of patients with chronic Kidney failure and DM2 in the cohorts included in this study. These populations have greater numbers of risk factors associated with death and cardiovascular events,^52^ among the non-invasive techniques used to evaluate arterial stiffness, which may have contributed to the number of events observed. Differences in the equipment used to estimate central pressure between studies may have allowed measurement bias. The different populations studied may also have contributed to the heterogeneity that was observed. However, these limitations do not invalidate the strong association observed in the present meta-analysis, in predicting future cardiovascular events and all-cause mortality in a strong and independent manner.

This systematic review and meta-analysis examined the relationship between central blood pressure measurements using different non-invasive techniques and occurrences of hard outcomes such as cardiovascular and all-cause mortality in a population over the age of 60 years. Most previous studies focused on younger populations and used intermediary outcomes. The present meta-analysis results point to more promising results from aPWV for predicting all-cause mortality, while baPWV and central SBP demonstrated more consistent results for evaluating cardiovascular mortality outcomes. Thus, the findings support the usage of central blood pressure as a risk predictor for hard outcomes in an older population. The data extracted originated from wide-ranging cohort studies in quality was evaluated, and give grounds for the idea that wider usage of central blood pressure measurement without limitation through patient age is important.

## CONCLUSIONS

This study revealed that there was a strong association between central blood pressure and both cardiovascular and all-cause mortality outcomes in an older population. These findings support the idea that hemodynamic overload and the consequent physiopathology of arterial stiffness involve central vessels. From this perspective, the findings of this study support the idea of wider usage of tools for central blood pressure measurement in various clinical scenarios, as an independent prognostic marker. This study also provides a stimulus towards production of further studies on the clinical impact of these findings.
